# Supplementary material for: Partisan self-interest is an important driver for people’s support for the regulation of targeted political advertising
Source: PLoS One. 2021 May 12;16(5):e0250506. doi: 10.1371/journal.pone.0250506 (PMC8115848; doi:10.1371/journal.pone.0250506)
Supplement: S1 File — (PDF) [file pone.0250506.s001.pdf]

S1 File

The supporting information provides additional background information (part A), names and explains deviations from the pre-analysis plan (part B), provides an overview of the experimental design and the instructions (part C) and presents additional results (part D).

A Additional text

S1 Text. Online targeted political advertising

Online targeted advertising refers to a kind of advertising where people's data is used to direct advertising content to them that maximizes the likelihood of them reacting to it [1]. Thus, online targeted advertisement is shown to people who most likely already have a preference for its content, which increases ad spending efficiency [2]. In case of online political targeting, this means that parties show advertisement to voters who they are most likely to mobilize. Furthermore, political actors can customize their messages to the distinctive interests and concerns of these people, addressing issues that matter to them and employing language and form that appeals to them [3]. Data used for targeting can be demographic, or contain information about attitudes, interests, or personality traits, which either originates from public data or commercial, that can be deduced from data that individuals revealed online [4, 5]. This practice of tailoring advertisement based on personality variables is called "psychographic profiling" and often makes use of research on decision making to influence the recipient's attitudes [6].

While collecting voter data for advertising purposes is not new, the vast amount of personal data used for targeting and tailoring political ads is unprecedented and online behavioral data plays a major role in this [7]. With people generating more and more trackable information while surfing online, this data has gained significant importance for advertisers [5]. Online behavioral data can include search and purchase histories, websites visited, articles read or videos watched and what people communicate in e-mails and on social media [8, 5]. This data is gathered and then resold by so called "commercial data brokers", or companies whose business is "collecting personal information about consumers from a variety of sources and aggregating, analyzing, and sharing that information, or information derived from it, for purposes such as marketing" [9]. These firms track online behavioral data by using tracking cookies, small text files that are stored on the user's computer after visiting a website. Tracking cookies that are placed on a website by another entity are also called third-party cookies, while cookies that originate from a website itself are called first-party cookies [7]. Third-party cookies can track users over several websites, with the data usually being used for advertising [5]. An individual website can have up to 350 built in cookies

and the 100 most popular websites have more than 6000 cookies combined, more than 80 % of them being third-party cookies [10]. Tracking cookies can trace users' browsing history (including text entered or buttons clicked) and set-up long-term records of their online behavior [10]. These personal user histories are then sold by the commercial data brokers to political strategists and are then combined with offline data from voter registration databases, response data from door-to-door canvassing, telephone surveys or online behavioral data collected via candidates' websites. Subsequently, they are merged into detailed voter profiles [4, 7]. Campaigners can then apply predictive modeling techniques to the data to make inferences on which users are most likely to vote for a party, and how to best address them [6].

S2 Text. The role of privacy concerns in the regulation of online targeted political advertising

A large body of literature has shown that individual privacy concerns are an important determinant for people's attitudes towards targeted advertising [11, 12, 13, 14, 15]. The results of these studies also reveal that individual privacy attitudes are closely linked to individual preferences towards stricter privacy regulation [16, 17, 18]. This literature has identified several reasons for this connection. People's individual risk preferences can play a role in determining their stance on stricter regulation. If people perceive that the collection of their data can create large risks to them, for example due to data breaches, they are more likely to support stricter government regulation [12, 19]. Further, trust towards internet companies and the firms collecting data is an important factor in determining people's attitudes towards regulation [20, 21]. If people have high levels of trust towards these companies they are less likely to support sweeping government regulation and believe that industry self-regulation is sufficient [22]. Additionally, people might see their private data as valuable commodity and demand stricter protection of their property right [19]. They therefore demand stricter government laws to ensure that their property right is upheld [23, 24]. Additionally, users might perceive targeted advertising as "creepy" when it relies on their private data too heavily [25, 26]. Taken together, these different motives lead to a heightened sense of concern among people and can therefore motivate them to support stricter government regulation.

S3 Text. The third-person effect

The third-person effect is broadly defined as people's perception that other people are influenced by undesirable mass communication to a larger extent than themselves [27]. The emergence of the third-person effect is linked to a more general perception that people see biases and mistakes more strongly in others than in themselves [28, 29]. Previous research also suggests that people

in general have too optimistic views of their own future outcomes and overestimate their abilities [30].

Undesirableness of media content is defined as having either socially unacceptable content or intent [31] and the third-person effect is most likely to emerge when the topic of the media content is of personal importance and is perceived to be negatively biased against people's own interest [32]. Whether media content is perceived as undesirable can be dependent on societal factors and might be influenced by cultural traditions [33]. Previous research has focused on media content that describes societal taboo topics like pornography [34], gambling [35], violence [36], unhealthy behaviors [37] or, more recently, fake news [38] and has reliably found a third-person effect in those circumstances. The strength of the third-person effect also depends on the social distance to the "other" [39]. The more dissimilar, the more people think that that person will be influenced by undesirable media messages. Other work has focused on socially desirable topics (like ones promoting healthy behavior [37] or disaster preparedness [40]) and have found the opposite effect, a first-person effect. A first-person effect is the perception that people themselves are more influenced by this sort of media content as compared to others. This is in line with people's belief that they themselves are more likely to make wise decisions and in general have higher abilities.

Previous research has further identified a second part of the third-person effect: The behavioral part. This is a direct consequence of people's perception of the media's effect on others. They adopt behaviors to rectify the consequences of undesirable media messages on others [41]. The perception that others are strongly influenced by media messages can trigger different responses, for example adaptive behavior [41]. This study focuses on behavior that is intended to rectify the consequences of undesirable media communication. This behavior involves the support for censorship of undesirable media content to shield more vulnerable groups from its influence [42]. Alternatively, it can also be driven by the intention to prevent negative influences for society, for example in the case of the promotion of unhealthy behavior [43]. Rectifying behavior can also, in certain cases, lead to behavior that limits access of vulnerable groups to media [44] or increase support for media literacy training [38].

Deviations from the pre-analysis plan

S4 Text. Deviations from the pre-analysis plan

The pre-analysis plan is available here: AEARCTR-0005296.

Data, full instructions, variable coding and analysis code are available at a public OSF-repository. We deviated from the pre-plan in minor ways. We excluded 41 responses on the survey because they were submitted from people that entered the survey multiple times. Because we did not expect the technical possibility that people can enter the survey several times, we did not consider that in our pre-analysis plan. None of our results is sensitive to the exclusion of these participants. We further had to exclude incomplete responses from some regressions. Our regression results show that the inclusion of control variables does not significantly affect our main parameters.

The regression we report in table 2 is pre-registered as secondary data analysis. The primary outcome is reported in the supporting information in table S4. Both results are in line with the hypothesis that we pre-registered.

We further did not pre-register the comparison between privacy concerns in treatment and control condition explicitly. We pre-registered more generally that we will compare attitudes between treatment and control condition.

We further pre-registered some tests as one-sided tests. We decided to report two-sided results for all tests for ease of interpretation of the reader. Analyses that were not pre-registered are reported as exploratory data-analysis in the main body of the paper.

B Experimental design and instructions

S1 Fig. Overview of the experimental design

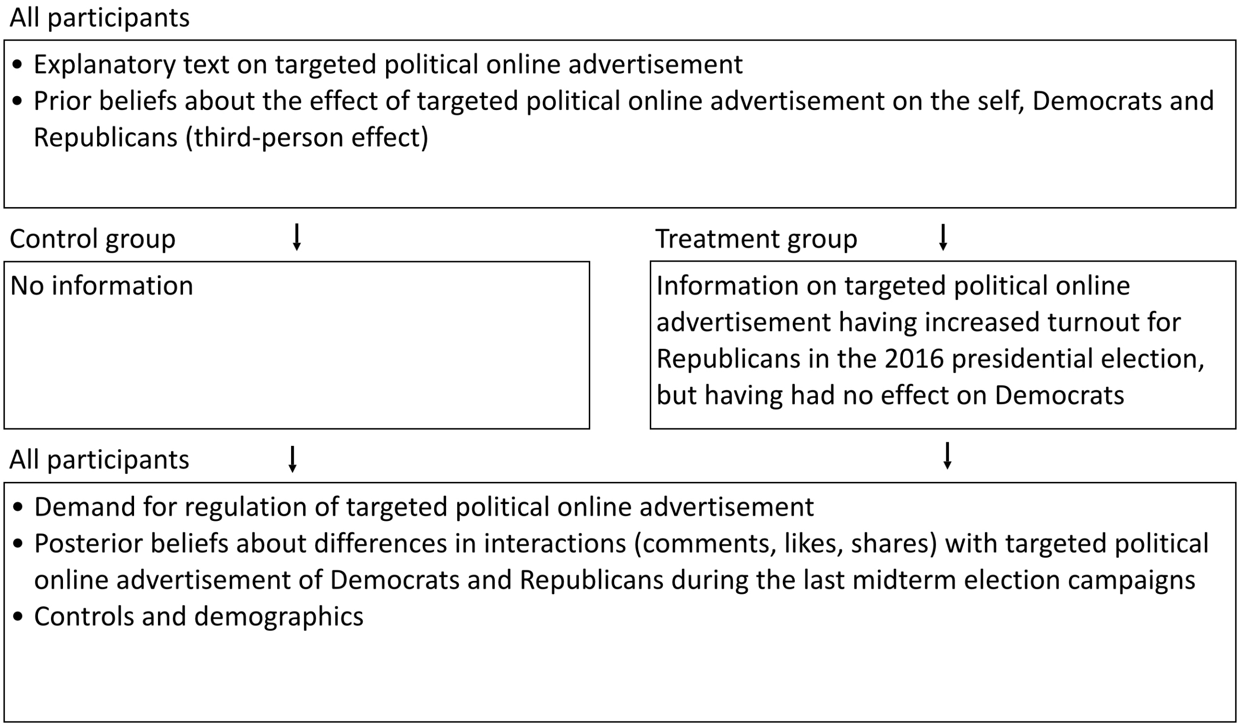

S2 Fig. Information about online targeted political advertising

Please read the following information carefully.

Targeted advertising is the practice of monitoring peoples' online behavior and using the collected information to show people individually targeted advertisements. Online behavioral data can include web browsing data, search histories, media consumption data (e.g., videos watched), app use data, purchases, click-through responses to ads, and communication content, such as what people post on social networking sites. This online data is often combined with demographic data like age, gender and location.

Political parties also use targeted advertising, for example before presidential elections. Targeted political advertisement involves creating messages targeted at narrow categories of voters based on data analysis gathered from individuals' demographic characteristics and their online behavior. This enables political campaigns to send very specific messages to certain groups of potential voters. These messages are selected to be the most appealing to this group. Political actors use targeted advertising, for example, to reach voters who are likely to vote for them with messages that will influence them.

I read and understood the information.

- ◯ Yes
- ◯ No

S3 Fig. Question about the belief on the effect of online targeted political advertising - Intro Screen

Both, Republicans and Democrats, use targeted political advertising on social media and other online platforms to show personalized messages to voters.

Imagine a political campaign in the run-up to a competitive election. Suppose that both parties, Republicans and Democrats, spend the same amount of money on social media and other online platforms to present targeted political advertisements to their likely voters. They use information on location, gender, education and political ideology to determine which messages they are displaying to these potential voters. Political ideology is inferred from peoples' online behavior like their "liked pages" on social media or their news consumption.

We now want to know what you believe about the effect that targeted political online advertising would have on potential voters.

Please answer the following questions to the best of your knowledge. This is important for the research project.

S4 Fig. Question about the belief on the effect of online targeted political advertising

To what extent do you think **targeted political online ads** have an influence on you?

- ◯ Not at all
- ◯ To a small extent
- ◯ To some extent
- ◯ To a great extent
- ◯ To a very great extent

To what extent do you think **targeted political online ads** have an influence on **Republicans?**

- ◯ Not at all
- ◯ To a small extent
- ◯ To some extent
- ◯ To a great extent
- ◯ To a very great extent

To what extent do you think **targeted political online ads** have an influence on **Democrats**?

- ◯ Not at all
- ◯ To a small extent
- ◯ To some extent
- ◯ To a great extent
- ◯ To a very great extent

S5 Fig. Treatment-Information

A group of international researchers investigated the influence of targeted political ads on voters. For their analysis, they looked at how many ads people saw and related that to increases in votes for the Republican and the Democratic parties. The ads were targeted based on peoples' location, gender, education, age and political ideology.

The researchers found that the targeted political ads significantly increased the number of votes for the Republican party, but not for the Democratic party. Hence, targeted political ads influenced Republican voters, but did not influence Democratic voters.

S6 Fig. Incentivisation - Message to Congress

We now want to know your opinion on certain policy initiatives that can be launched by the US government.

We will inform the members of the US Congress with an anonymised summary of the attitudes towards these policy initiatives stated by all participants in the following questions. **There is no deception in this study. We will actually send a message with the results to US House Representatives and Senate members. However, your answers will remain anonymous. No one, not even the researchers involved in this study, will be able to match your answers to you.**

On the next page, you see a representation of what this message will look like.

S7 Fig. Measurement of support for regulation

To what extent do you agree with the following statements?

	Strongly disagree	Disagree	Somewhat disagree	Neither agree nor disagree	Somewhat agree	Agree	Strongly agree
Targeted political online advertising should be banned.	○	○	○	○	○	○	○
I support legislation that requires targeted political online advertising to be clearly marked as targeted.	○	○	○	○	○	○	○
More regulation is needed when it comes to targeted political online advertising.	○	○	○	○	○	○	○
The government is already doing enough to regulate targeted political online advertising.	○	○	○	○	○	○	○

S8 Fig. Measurement of posterior beliefs

In this question, you can earn a small bonus based on the accuracy of your answer.

During the 2018 midterm elections, both major parties, Republicans and Democrats, used targeted advertising on social media platforms with the aim of influencing voters. One way to measure the success of this strategy is to see how much people interact (like, share, comment) with these advertising messages.

In the run-up to the 2018 elections, an international company measured interactions of people with Facebook posts of candidates for the House of Representatives. The measurement was taken between the 15. September and the 15. October 2018.

Please indicate now what you believe about the number of interactions that parties received relative to each other on these posts. If your answer is equal to the (rounded) correct answer, you will receive a bonus of $1.

- ◯ Democrats received four times the amount of Republicans
- ◯ Democrats received three times the amount of Republicans
- ◯ Democrats received twice the amount of Republicans
- ◯ Both received about the same amount
- ◯ Republicans received twice the amount of Democrats
- ◯ Republicans received three times the amount of Democrats
- ◯ Republicans received four times the amount of Democrats

S9 Fig. Measurement of privacy concerns

To what extent do you agree with the following statements?
On the internet, I am concerned that my information:

	Strongly agree	Agree	Somewhat agree	Neither agree nor disagree	Somewhat disagree	Disagree	Strongly disagree
...can be collected and stored by third parties.	◯	◯	◯	◯	◯	◯	◯
...can be shared with other third-parties (e.g. advertisers, employer, state).	◯	◯	◯	◯	◯	◯	◯
Please choose "strongly agree" for this statement.	◯	◯	◯	◯	◯	◯	◯
...can be used to display targeted advertising to me.	◯	◯	◯	◯	◯	◯	◯
...can be used for commercial purposes (e.g. targeted advertising).	◯	◯	◯	◯	◯	◯	◯

C Additional figures and results

S10 Fig. Density plot of participants' support for stricter regulation of online targeted political advertising

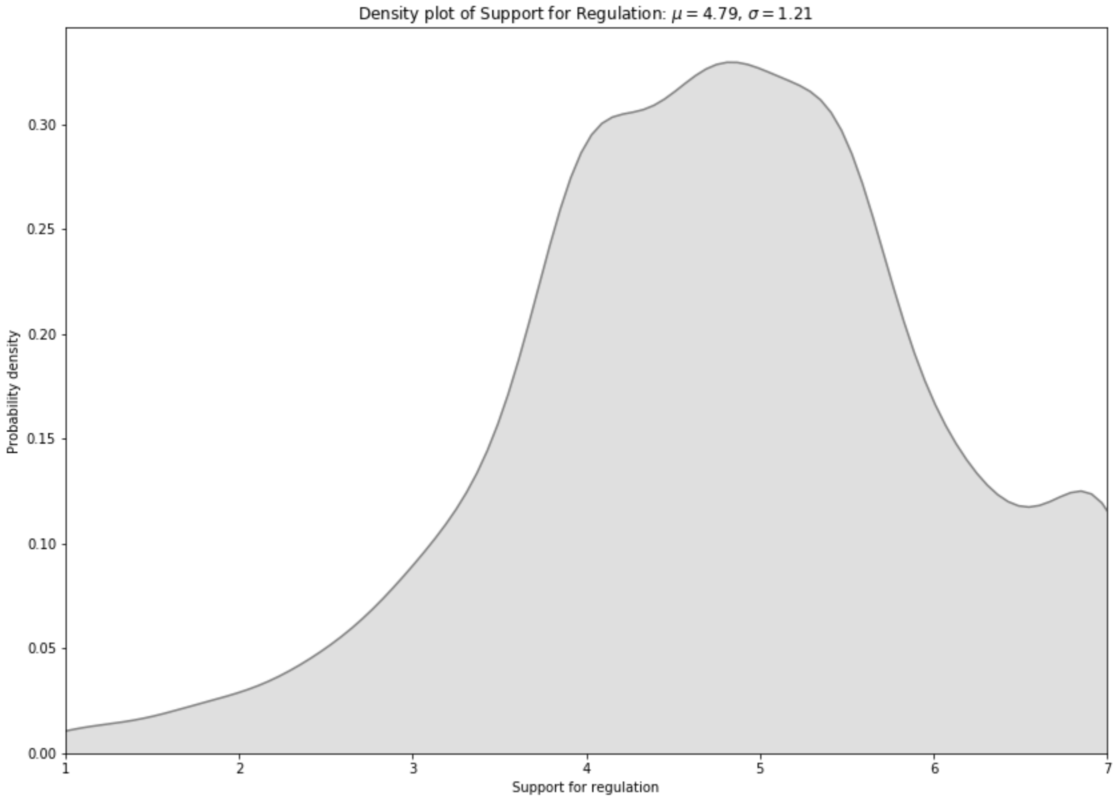

Note: Support for regulation was measured on a four item, seven-point Likert Scale. The plot shows the distribution of support for regulation for all participants

S11 Fig. Density plot of participants' privacy concerns

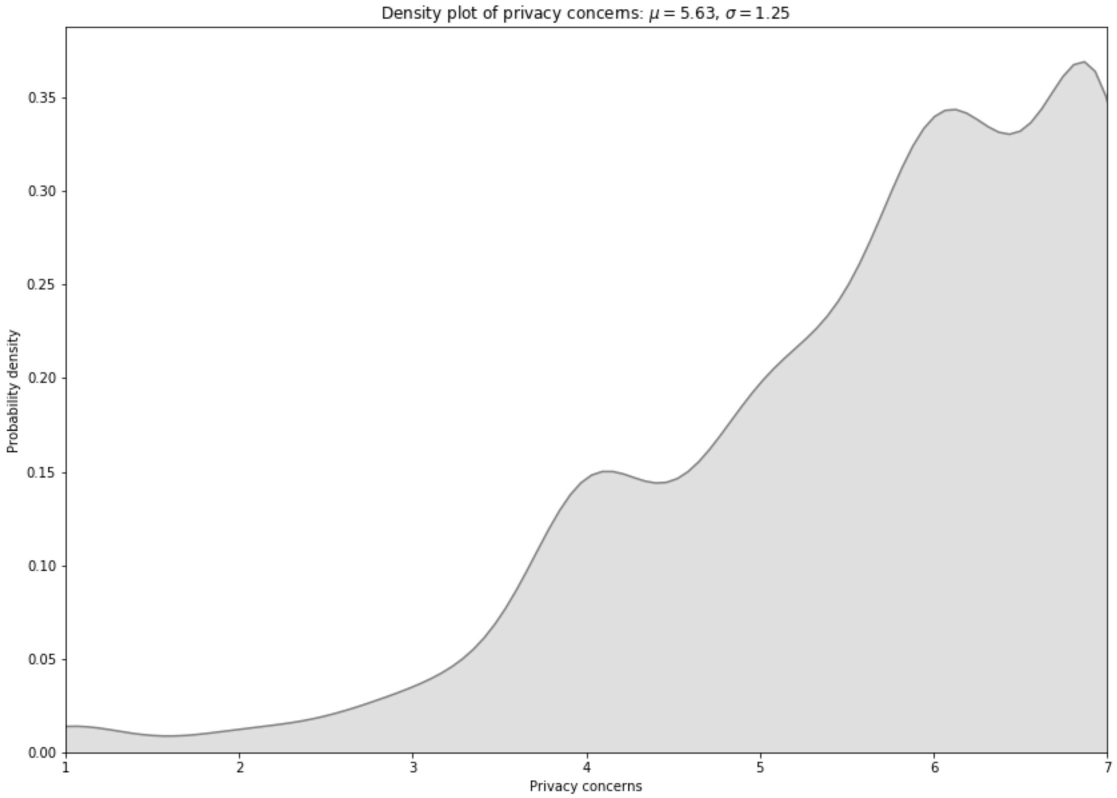

Note: Privacy concerns were measured on a four item, seven-point Likert Scale. The plot shows the distribution of privacy concerns for all participants

S12 Fig. Relationship prior beliefs and posterior beliefs for participants in the control group

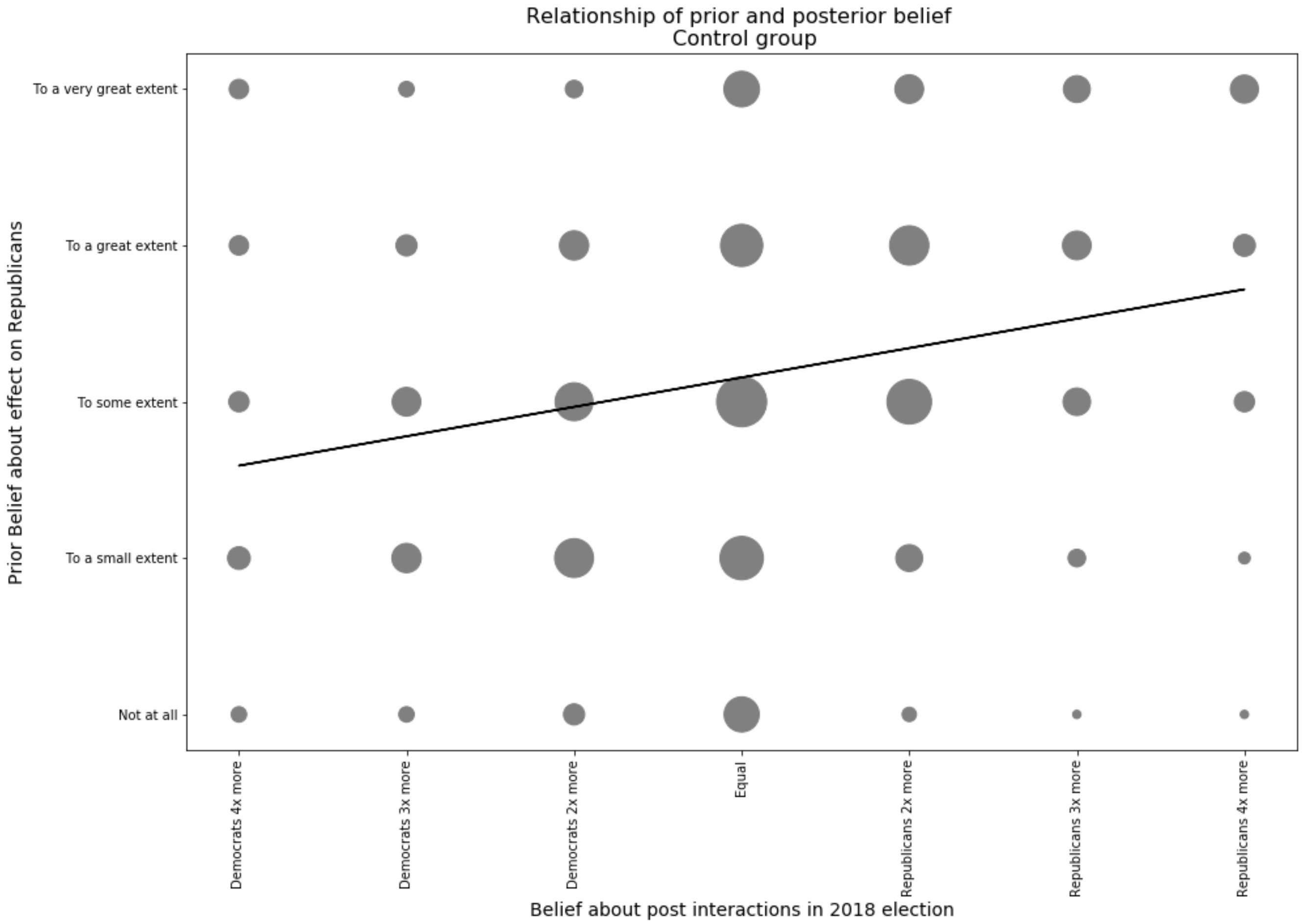

Note: The size of the circles indicate frequency of combinations. If the circle is bigger that indicates a higher frequency of both answers applying. The line shows a linear regression between the two measures.

S13 Fig. Prior and posterior beliefs, Republicans

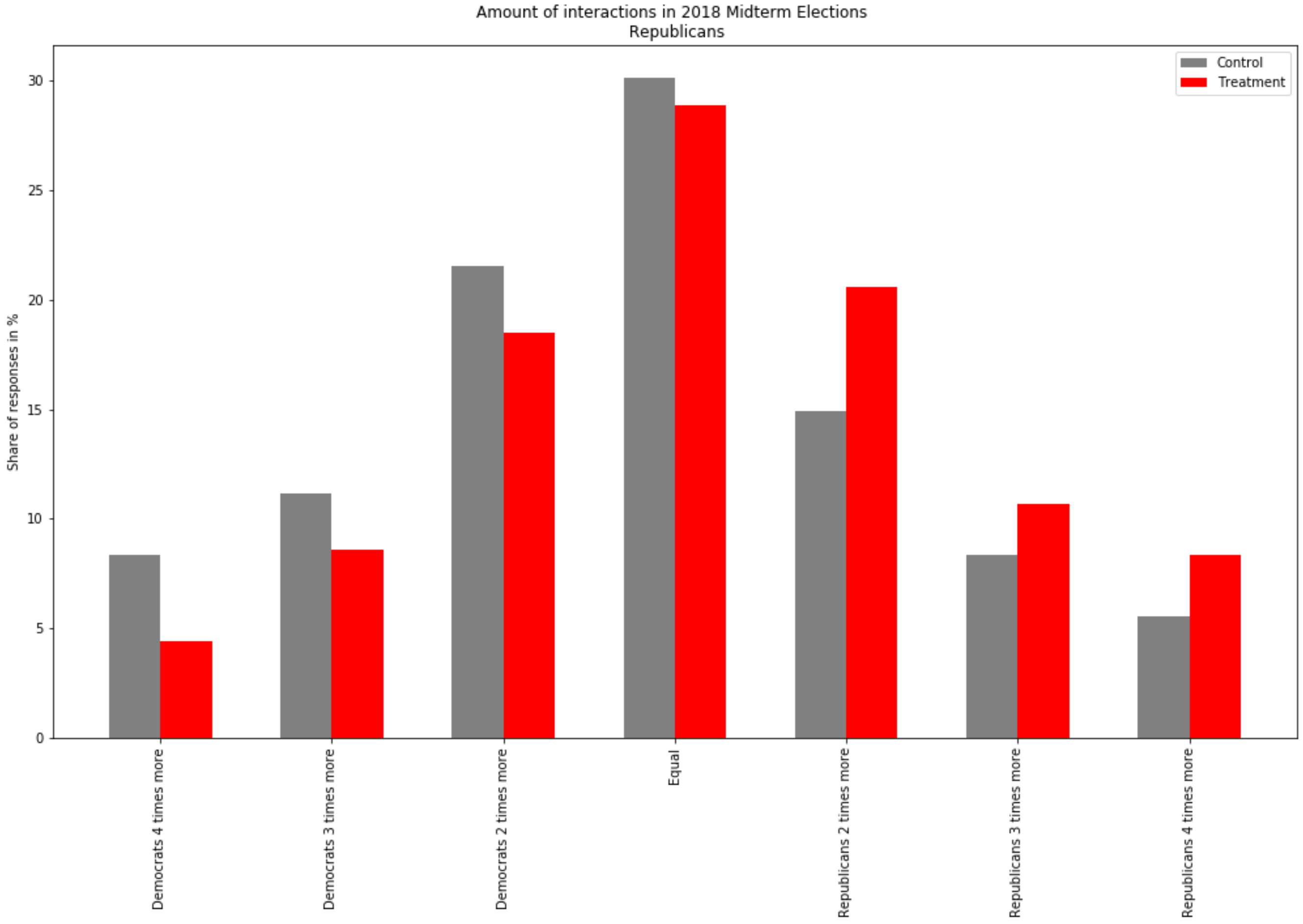

Note: The figure shows the responses of Republicans to the question displayed in figure 10. The grey bars indicate responses for participants in the control condition. The red bars indicate responses in the treatment condition.

S14 Fig. Prior and posterior beliefs, Democrats

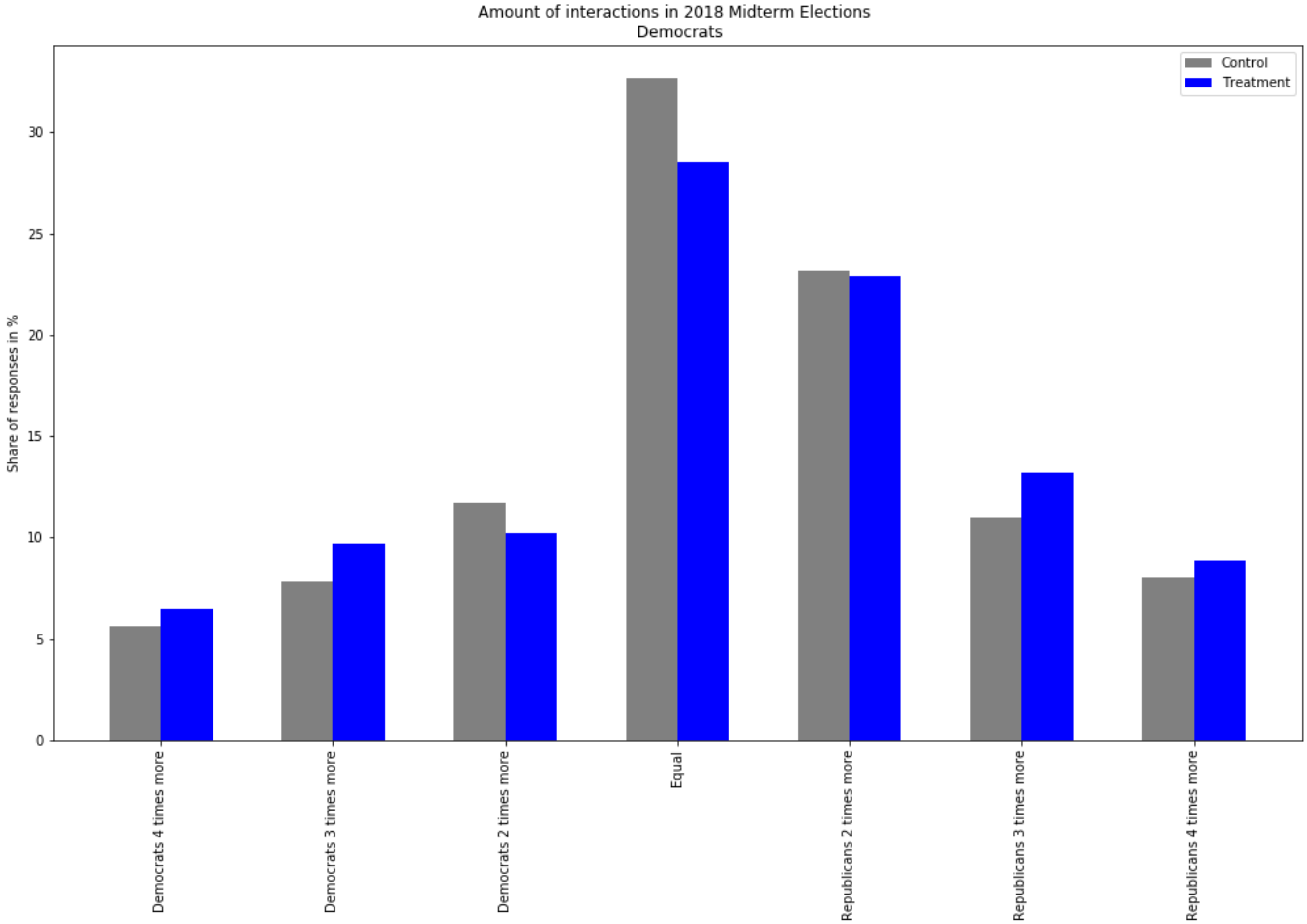

Note: The figure shows the responses of Democrats to the question displayed in figure 10. The grey bars indicate responses for participants in the control condition. The blue bars indicate responses in the treatment condition.

S15 Fig. Treatment effect, Republicans

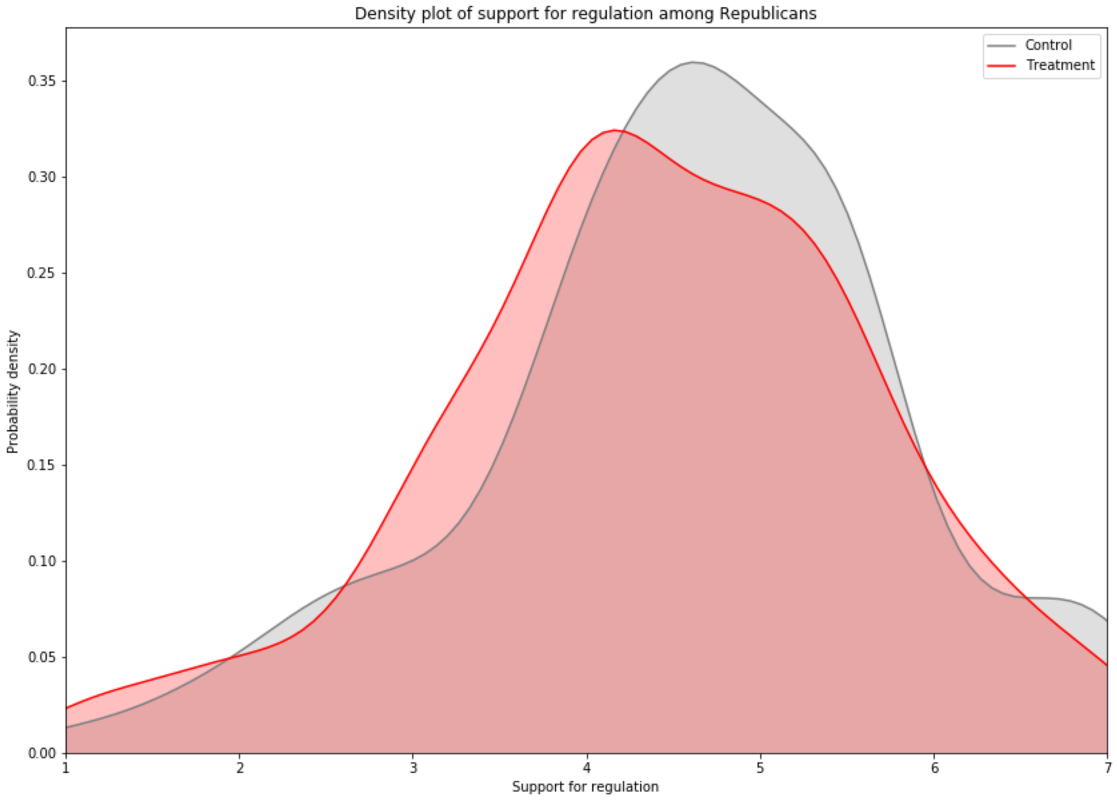

Note: The figure displays support for stricter government regulation among Republicans. Support for regulation was measured on a four item, seven-point Likert Scale. The red area indicates the distribution for Republicans in the treatment condition. The grey area displays the distribution for Republicans in the control condition.

S16 Fig. Treatment effect, Democrats

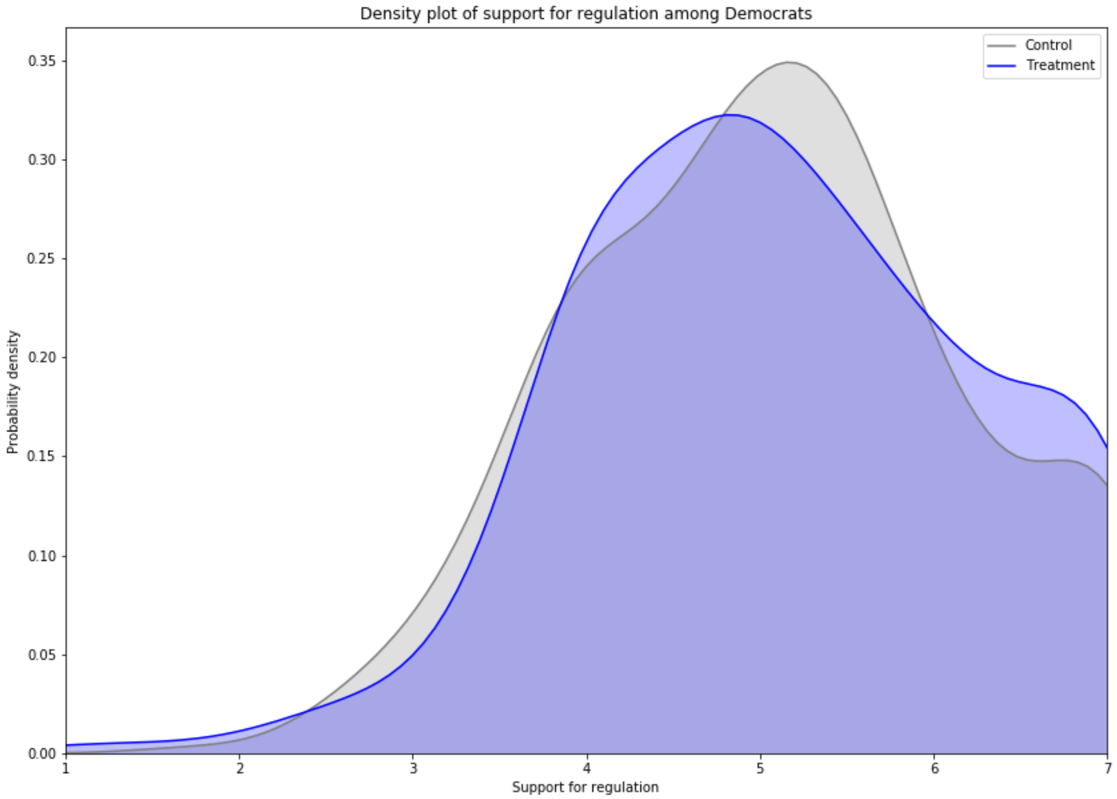

Note: The figure displays support for stricter government regulation among Democrats. Support for regulation was measured on a four item, seven-point Likert Scale. The blue area indicates the distribution for Democrats in the treatment condition. The grey area displays the distribution for Democrats in the control condition.

S17 Fig. Treatment effect, Republicans below median conservative

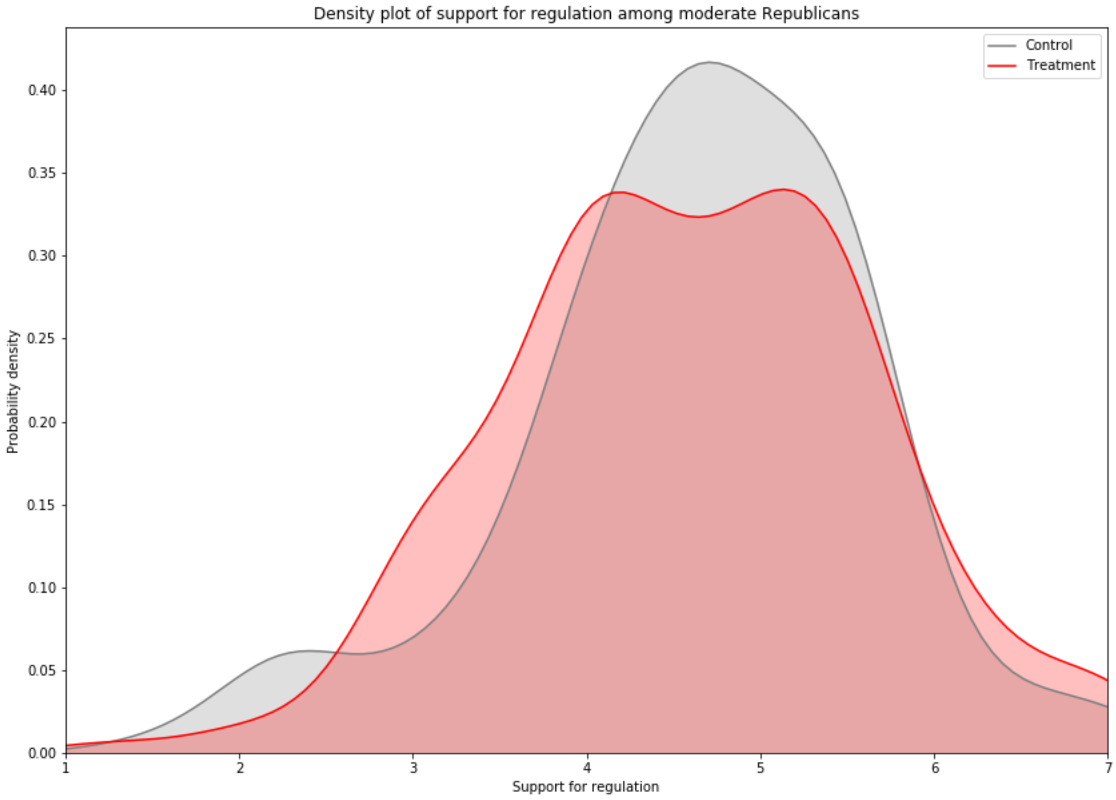

Note: The figure displays support for stricter government regulation among moderate Republicans. Support for regulation was measured on a four item, seven-point Likert Scale. Political ideology was measured on a scale for economic and social conservatism. Moderate Republicans were defined as being below median in this measure. The red area indicates the distribution for moderate Republicans in the treatment condition. The grey area displays the distribution for moderate Republicans in the control condition.

S18 Fig. Treatment effect, Republicans at or above median conservative

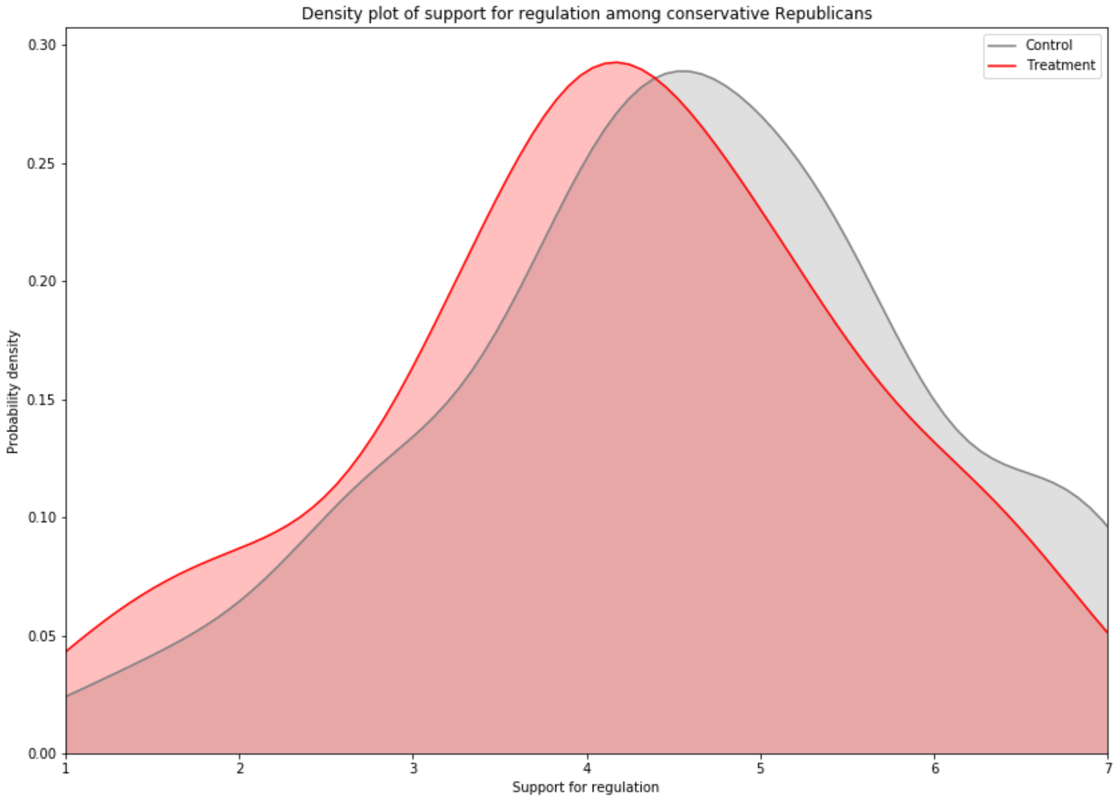

Note: The figure displays support for stricter government regulation among moderate Republicans. Support for regulation was measured on a four item, seven-point Likert Scale. Political ideology was measured on a scale for economic and social conservatism. Conservative Republicans were defined as being at or above median in this measure. The red area indicates the distribution for conservative Republicans in the treatment condition. The grey area displays the distribution for conservative Republicans in the control condition.

S1 Table. Descriptive Statistics

	Number of participants	Share
Income lower than $30,000	289	0.19
Income between $30,000 and $60,000	426	0.28
Income between $60,000 and $100,000	392	0.25
Income between $100,000 and $140,000	174	0.11
Income higher than $140,000	161	0.10
Less than high school	19	0.01
High school/GED	262	0.17
Some college	322	0.21
Associate degree	185	0.12
Bachelor degree	406	0.26
Postgraduate degree	287	0.19
Non-white participants	388	0.25
User of social media	1359	0.88
Observations	1549	

Note: The table provides an overview of demographic variables for all participants in the experiment.

S2 Table. Descriptive Statistics, group comparisons

	Control group		Treatment group	
	Share		Share	
Income lower than $30,000	0.18		0.19	
Income between $30,000 and $60,000	0.28		0.27	
Income between $60,000 and $100,000	0.26		0.24	
Income between $100,000 and $140,000	0.11		0.11	
Income higher than $140,000	0.10		0.11	
Less than high school	0.01		0.01	
High school/GED	0.16		0.18	
Some college	0.23		0.19	
Associate degree	0.11		0.13	
Bachelor degree	0.27		0.25	
Postgraduate degree	0.18		0.19	
Non-white participants	0.25		0.25	
Social media user	0.89		0.87	
	Mean	SD	Mean	SD
Age	47.49	16.487	47.60	16.137
Belief about effect of the ads on self	2.38	1.208	2.41	1.213
Belief about the effect of the ads on Democrats	3.07	1.111	3.08	1.110
Belief about the effect of the ads on Republicans	3.17	1.201	3.07	1.136
Privacy concerns	5.60	1.261	5.67	1.254
Observations	794		755	

Note: The table provides an overview of demographic variables for participants split into treatment and control group in the experiment.

S3 Table. Regression of determinants predicting the size of the difference between the perceived effect of the ads on the other party versus the own party

	Coef.	Robust SE	p-value	95% CI
Affective polarization	0.170	0.032	<0.001	0.107, 0.232
Ideological polarization	0.112	0.031	<0.001	0.051, 0.174
Desirability of advertising	-0.149	0.017	<0.001	-0.183, -0.116
High political knowledge	0.133	0.065	0.040	0.006, 0.261
High income	-0.087	0.065	0.184	-0.241, 0.041
Education	0.076	0.064	0.236	0.050, 0.201
Male	-0.064	0.062	0.304	-0.185, 0.058
Non-white participants	-0.091	0.074	0.222	-0.236, 0.055
Age	0.004	0.002	0.088	-0.001, 0.008
Household size	-0.009	0.065	0.894	-0.135, 0.118
Use of internet in hours	0.003	0.005	0.642	-0.008, 0.132
Use of ad-block	0.018	0.029	0.530	-0.039, 0.076
User of social media	0.000	0.085	0.995	-0.166, 0.167
Attitude towards government regulation	-0.013	0.016	0.424	-0.046, 0.019
External efficacy	-0.002	0.001	0.119	-0.004, 0.001
Politically active	0.078	0.060	0.193	-0.039, 0.197
Constant	0.808	0.197	<0.001	0.422, 1.193
Observations	1464			
R^2	0.148			

Note: The table reports the results for an OLS-regression with the difference between how much participants thought online targeted political advertising influences voters of the other party minus how much they thought it influences voters of their own party as dependent variable. The dependent variable is standardized. Affective and ideological polarization are standardized. High income is a dummy for above median in the sample, education is a dummy for above median in the sample, male is a dummy for being male, and non-white is a dummy for being non-white, household size is a dummy for more than two members. User of social media is a dummy for the use of social media, use of ad-block is a dummy for ad-block use. Political engagement is a dummy variable for being politically active within the last year, political knowledge is a dummy for above median knowledge.

S4 Table: Regression of determinants for the willingness to support stricter regulation of online targeted political advertising, control group

	Coef.	Robust SE	p-value	95% CI
Belief about own party	-0.010	0.048	0.837	-0.105, 0.847
Belief about other party	0.172	0.039	<0.001	0.095, 0.249
Belief about self	-0.019	0.046	0.681	-0.108, 0.071
Privacy concerns	0.239	0.045	<0.001	0.151, 0.326
Observations	754	754		
R^2	0.115	0.135		
Demographics	No	Yes		
social media use	No	Yes		
Political Engagement	No	Yes		

Note: Regressions only include participants in the control group who answered all questions of the survey. The table reports results from an OLS-regression with people's support for regulation as the dependent variable. The variable is standardized. Belief other party is measured as participants' belief about the effect that online targeted political advertisement has on supporters of the other party. Belief own party is defined as the belief that participants have about the effect online targeted political advertising has on supporters of their own party. Belief about self is people's belief about the effect that online targeted political advertising has on themselves. Privacy concerns are measured on a seven-point Likert scale (SA - SD). The variable is standardized. Demographic information includes age, education, income, household size, gender, and ethnicity. Social media use includes whether the participant uses social media, the time they spend online in general (in hours), and and the use of an ad-blocker. Political engagement measures include a variable for being politically active within the last year, external political efficacy, political knowledge, and attitudes towards government regulation in general. S9 Table provides an overview of all variables in the regression.

S5 Table. Effect of the information treatment on Republicans, Republicans who believe treatment information only

	Baseline	Treatment	Difference
Observations	391	315	
Support for regulation	4.59	4.47	-0.12

Demand for regulation measured on a four item 7 point Likert scale. p-value = 0.10 for one-sided t-test

Note: The table summarizes a Welsh-t-test that is run to compare Republicans' support for regulation. Support for regulation was measured on a seven-point Likert scale (SA - SD). Only Republicans who trust the presented research finding were included. The p-value of that test was 0.10 for a one-sided t-test. * $p < 0.05$, ** $p < 0.01$, *** $p < 0.001$

S6 Table. Effect of the information treatment on Republicans - Only Republicans who stated they want Congress to take their answers into account

	Baseline	Treatment	Difference
Observations	388	381	
Demand for regulation	4.58	4.41	-0.17*

* $p < 0.05$, ** $p < 0.01$, *** $p < 0.001$

Demand for regulation measured on a four item 7 point Likert scale. p-value = 0.03 for one-sided t-test

Note: The table summarizes a Welsh-t-test that is run to compare Republicans' support for regulation. Support for regulation was measured on a seven-point Likert scale (SA - SD). Only Republicans who want their answers to be taken into account by Congress are included. The p-value of that test was 0.03 for a one-sided t-test. * $p < 0.05$, ** $p < 0.01$, *** $p < 0.001$

S7 Table. Regression of determinants for the willingness to support stricter regulation of targeted advertising - Alternative regulation measure

	Coef.	Robust SE	p-value	95% CI
Belief other party - own party	0.107	0.036	0.003	0.037, 0.176
Privacy concerns	0.262	0.043	<0.001	0.178, 0.347
Belief about self	0.116	0.039	0.003	0.040, 0.193
Observations	754			
R^2	0.161			
Demographics	Yes			
Social media use	Yes			
Political Engagement	Yes			

The regression only includes participants who answered all questions of the survey. The table reports results from an OLS-regression in which people's support for regulation is the dependent variable. The fourth item was excluded from the scale. The value is standardized. Belief other party-own party is defined as the difference between people's belief about the effect on the other party and the effect on the own party. Belief about self is people's belief about the effect that online targeted political advertising has on them. Privacy concerns are respondents' are measured on a seven-point Likert scale (SA - SD). All three independent variables are standardized. Demographic information includes age, education, income, household size, gender, and ethnicity. Social media use includes whether the participant uses social media, the time they spend online in general (in hours), and and the use of an ad-blocker. Political engagement measures include a variable for being politically active within the last year, external political efficacy, political knowledge, and attitudes towards government regulation in general.

S8 Table. Regression of Determinants for the willingness to regulate targeted ads - Alternative regulation measure

	Coef.	Robust SE	p-value	95% CI
Treatment*Republican	-0.252	0.103	0.014	-0.454, -0.051
Treatment	0.099	0.066	0.132	-0.030, 0.223
Republican	-0.204	0.071	0.004	-0.344, -0.064
Observations	1466			
R^2	0.031			

Note: Regressions only include participants who answered all questions of the survey. The table reports the results of an OLS-regression with the support for regulation of online targeted political advertising as a dependent variable. The variable was standardized. Treatment is a dummy variable that is 1 when the participant was assigned to the treatment condition and 0 otherwise. Republican is a dummy variable that is 1 when the participant is a Republican and 0 if he or she is a Democrat. Treatment*Republican is the interaction of these two variables. Privacy concerns are measured on a seven-point Likert scale (SD - SA). The variable is standardized.

S9 Table. Regression of determinants for the willingness to support stricter regulation of targeted advertising - Alternative regulation measure 2

	Coef.	Robust SE	p-value	95% CI
Belief other party - own party	0.116	0.039	0.003	0.040, 0.193
Privacy concerns	0.287	0.047	<0.001	0.194, 0.379
Belief about self	0.127	0.043	0.003	0.044, 0.210
Observations	754			
R^2	0.161			
Demographics	Yes			
Social media use	Yes			
Political Engagement	Yes			

The regression only includes participants who answered all questions of the survey. The table reports results from an OLS-regression in which people's support for regulation is the dependent variable. The fourth item was excluded from the scale. The value is standardized. Belief other party-own party is defined as the difference between people's belief about the effect on the other party and the effect on the own party. Belief about self is people's belief about the effect that online targeted political advertising has on them. Privacy concerns are respondents' are measured on a seven-point Likert scale (SA - SD). All three independent variables are standardized. Demographic information includes age, education, income, household size, gender, and ethnicity. Social media use includes whether the participant uses social media, the time they spend online in general (in hours), and and the use of an ad-blocker. Political engagement measures include a variable for being politically active within the last year, external political efficacy, political knowledge, and attitudes towards government regulation in general.

S10 Table. Regression of Determinants for the willingness to regulate targeted ads - Alternative regulation measure 2

	Coef.	Robust SE	p-value	95% CI
Treatment*Republican	-0.276	0.112	0.014	-0.496, -0.055
Treatment	0.109	0.072	0.132	-0.033, 0.250
Republican	-0.223	0.078	0.004	-0.376, -0.070
Observations	1466			
R^2	0.031			

Note: Regressions only include participants who answered all questions of the survey. The table reports the results of an OLS-regression with the support for regulation of online targeted political advertising as a dependent variable. The variable was standardized. Treatment is a dummy variable that is 1 when the participant was assigned to the treatment condition and 0 otherwise. Republican is a dummy variable that is 1 when the participant is a Republican and 0 if he or she is a Democrat. Treatment*Republican is the interaction of these two variables. Privacy concerns are measured on a seven-point Likert scale (SD - SA). The variable is standardized.

S11 Table. Regression of determinants for the willingness to support stricter regulation of online targeted political advertising, control group, all control variables

	Coef.	Robust SE	p-value	95% CI
Belief other party - own party	0.124	0.035	<0.001	0.551, 0.193
Privacy concerns	0.257	0.045	<0.001	0.169, 0.344
Belief about self	0.052	0.039	0.187	-0.025, 0.129
High income	0.049	0.073	0.502	-0.095, 0.193
Education	-0.058	0.075	0.438	-0.206, 0.090
Male	-0.112	0.070	0.110	-0.249, 0.025
Non-white participants	-0.142	0.076	0.062	-0.292, 0.007
Age	0.003	0.002	0.200	-0.002, 0.008
Household size	0.063	0.073	0.388	-0.080, 0.206
Use of internet in hours	-0.002	0.006	0.689	-0.014, 0.009
Use of ad-block	-0.062	0.035	0.079	-0.131, 0.007
User of social media	0.149	0.128	0.247	-0.103, 0.400
Attitude towards government regulation	0.017	0.018	0.362	-0.019, 0.052
High political knowledge	0.017	0.079	0.833	-0.139, 0.172
External efficacy	-0.001	0.001	0.221	-0.004, 0.001
Politically active	-0.054	0.085	0.527	-0.221,0.113
Constant	-0.132	0.235	0.574	-0.593, 0.329
Observations	754			
R^2	0.125			

Note: The table reports results for the OLS-regression support for regulation of online targeted political advertising as the dependent variable. Results are reported for participants in the control condition who answered all questions. All non-control variables are standardized. Belief other party-own party is defined as the difference between people's belief about the effect of online targeted political advertising on the other party minus its effect on the own party. Belief about self is people's belief about the effect that online targeted political advertising has on themselves. Privacy concerns are measured on a seven-point Likert scale (SA - SD). Education is a dummy for above median in the sample, income is a dummy for above median in the sample, household size is a dummy for more than two members, male is a dummy for being male, and non-white is a dummy for being non-white. User of social media is a dummy for the use of social media, use of ad-block is a dummy for ad-block use. Political engagement is a dummy variable for being politically active within the last year, political knowledge is a dummy for above median knowledge.

References

[1] Chen J, Stallaert J. An economic analysis of online advertising using behavioral targeting. Mis Quarterly. 2014;38(2):429–450.

[2] Iyer G, Soberman D, Villas-Boas JM. The targeting of advertising. Marketing Science. 2005;24(3):461–476.

[3] Zuiderveen Borgesius F, Möller J, Kruikemeier S, Ó Fathaigh R, Irion K, Dobber T, et al. Online political microtargeting: promises and threats for democracy. Utrecht Law Review. 2018;14(1):82–96.

[4] Hersh ED. Hacking the electorate: How campaigns perceive voters. Cambridge University Press; 2015.

[5] Boerman SC, Kruikemeier S, Zuiderveen Borgesius FJ. Online behavioral advertising: A literature review and research agenda. Journal of advertising. 2017;46(3):363–376.

[6] Burkell J, Regan PM. Voter preferences, voter manipulation, voter analytics: policy options for less surveillance and more autonomy. Internet Policy Review. 2019;8(4):1–24.

[7] Rubinstein IS. Voter privacy in the age of big data. Wis L Rev. 2014;5:861–936.

[8] Jansen BJ, Moore K, Carman S. Evaluating the performance of demographic targeting using gender in sponsored search. Information Processing & Management. 2013;49(1):286–302.

[9] Commission FT. Data brokers: a call for transparency and accountability. Report, FTC, Washington, DC. 2014;.

[10] Altaweel I, Good N, Hoofnagle CJ. Web privacy census. Technology Science. 2015;.

[11] Wang H, Lee MK, Wang C. Consumer privacy concerns about Internet marketing. Communications of the ACM. 1998;41(3):63–70.

[12] Okazaki S, Li H, Hirose M. Consumer privacy concerns and preference for degree of regulatory control. Journal of advertising. 2009;38(4):63–77.

[13] Baek TH, Morimoto M. Stay away from me. Journal of advertising. 2012;41(1):59–76.

[14] Evans DS. The online advertising industry: Economics, evolution, and privacy. Journal of economic perspectives. 2009;23(3):37–60.

[15] Krasnova H, Günther O, Spiekermann S, Koroleva K. Privacy concerns and identity in online social networks. Identity in the Information Society. 2009;2(1):39–63.

[16] Milberg SJ, Burke SJ, Smith HJ, Kallman EA. Values, personal information privacy, and regulatory approaches. Communications of the ACM. 1995;38(12):65–74.

[17] Smith HJ, Milberg SJ, Burke SJ. Information privacy: measuring individuals' concerns about organizational practices. MIS quarterly. 1996;20(2):167–196.

[18] Milberg SJ, Smith HJ, Burke SJ. Information privacy: Corporate management and national regulation. Organization science. 2000;11(1):35–57.

[19] Miltgen CL, Smith HJ. Exploring information privacy regulation, risks, trust, and behavior. Information & Management. 2015;52(6):741–759.

[20] Tang Z, Hu Y, Smith MD. Gaining trust through online privacy protection: Self-regulation, mandatory standards, or caveat emptor. Journal of Management Information Systems. 2008;24(4):153–173.

[21] Edelman B. Adverse selection in online "trust" certifications and search results. Electronic Commerce Research and Applications. 2011;10(1):17–25.

[22] Xu H, Dinev T, Smith J, Hart P. Information privacy concerns: Linking individual perceptions with institutional privacy assurances. Journal of the Association for Information Systems. 2011;12(12):798–824.

[23] Jentzsch N. The regulation of financial privacy: the United States Vs Europe. ECRI RESEARCH REPORT NO. 5; 2003.

[24] Acquisti A, Taylor C, Wagman L. The economics of privacy. Journal of economic Literature. 2016;54(2):442–92.

[25] Tene O, Polonetsky J. A theory of creepy: technology, privacy and shifting social norms. Yale JL & Tech. 2013;16:59–102.

[26] Moore RS, Moore ML, Shanahan KJ, Mack B. Creepy marketing: Three dimensions of perceived excessive online privacy violation. Marketing Management. 2015;25(1):42–53.

[27] Davison WP. The third-person effect in communication. Public opinion quarterly. 1983;47(1):1–15.

[28] Pronin E, Lin DY, Ross L. The bias blind spot: Perceptions of bias in self versus others. Personality and Social Psychology Bulletin. 2002;28(3):369–381.

[29] Scopelliti I, Morewedge CK, McCormick E, Min HL, Lebrecht S, Kassam KS. Bias blind spot: Structure, measurement, and consequences. Management Science. 2015;61(10):2468–2486.

[30] Sharot T. The optimism bias. Current biology. 2011;21(23):R941–R945.

[31] Gunther AC, Mundy P. Biased optimism and the third-person effect. Journalism Quarterly. 1993;70(1):58–67.

[32] Perloff RM. Third-person effect research 1983–1992: A review and synthesis. International Journal of Public Opinion Research. 1993;5(2):167–184.

[33] Paul B, Salwen MB, Dupagne M. The third-person effect: A meta-analysis of the perceptual hypothesis. Mass Communication & Society. 2000;3(1):57–85.

[34] Lo Vh, Wei R. Third-person effect, gender, and pornography on the lnternet. Journal of Broadcasting & Electronic Media. 2002;46(1):13–33.

[35] Youn S, Faber RJ, Shah DV. Restricting gambling advertising and the third-person effect. Psychology & Marketing. 2000;17(7):633–649.

[36] McLeod DM, Eveland Jr WP, Nathanson AI. Support for censorship of violent and misogynic rap lyrics: An analysis of the third-person effect. Communication Research. 1997;24(2):153–174.

[37] Henriksen L, Flora JA. Third-person perception and children: Perceived impact of pro-and anti-smoking ads. Communication Research. 1999;26(6):643–665.

[38] Jang SM, Kim JK. Third person effects of fake news: Fake news regulation and media literacy interventions. Computers in Human Behavior. 2018;80:295–302.

[39] Eveland Jr WP, Nathanson AI, Detenber BH, McLeod DM. Rethinking the social distance corollary: Perceived likelihood of expsoure and the third-person perception. Communication Research. 1999;26(3):275–302.

[40] Atwood LE. Illusions of media power: The third-person effect. Journalism Quarterly. 1994;71(2):269–281.

[41] Sun Y, Shen L, Pan Z. On the behavioral component of the third-person effect. Communication Research. 2008;35(2):257–278.

[42] Rojas H, Shah DV, Faber RJ. For the good of others: Censorship and the third-person effect. International Journal of Public Opinion Research. 1996;8(2):163–186.

[43] Shah DV, Faber RJ, Youn S. Susceptibility and severity: Perceptual dimensions underlying the third-person effect. Communication Research. 1999;26(2):240–267.

[44] Hoffner C, Buchanan M. Parents' responses to television violence: The third-person perception, parental mediation, and support for censorship. Media Psychology. 2002;4(3):231–252.
